# Fatostatin ameliorates inflammation without affecting cell viability

**DOI:** 10.1002/2211-5463.13364

**Published:** 2022-02-01

**Authors:** Shuhe Ma, Kosaku Murakami, Kazune Tanaka, Motomu Hashimoto, Masao Tanaka, Koji Kitagori, Shuji Akizuki, Ran Nakashima, Hajime Yoshifuji, Koichiro Ohmura, Akio Morinobu, Tsuneyo Mimori

**Affiliations:** ^1^ Department of Rheumatology and Clinical Immunology Kyoto University Graduate School of Medicine Kyoto Japan; ^2^ Center for Cancer Immunotherapy and Immunobiology Kyoto University Graduate School of Medicine Kyoto Japan; ^3^ Department for Advanced Medicine for Rheumatic Disease Kyoto University Graduate School of Medicine Kyoto Japan; ^4^ Department of Clinical Immunology Osaka City University Graduate School of Medicine Osaka Japan; ^5^ Ijinkai Takeda General Hospital Kyoto Japan

**Keywords:** cytokine, fatostatin, inflammation, lipid, SREBP

## Abstract

The mature form of sterol regulatory element‐binding protein (SREBP)1 is a transcription factor involved in lipid synthesis, which participates in toll like receptor 4‐triggered inflammatory pathways during the resolution phase of inflammation in macrophages. SREBP1 has thus attracted interest as a candidate target molecule for ameliorating inflammation. Fatostatin is a small molecule that inhibits the maturation and function of SREBP, and its role in regulating inflammation is poorly understood. To evaluate the anti‐inflammatory effect of fatostatin, we compared body weight, footpad and hock dimensions, and arthritis scores between K/BxN serum‐induced arthritis mice treated with fatostatin and those treated with dimethyl sulfoxide as the vehicle control. We performed hematoxylin and eosin staining of joints of distal paws to assess tissue inflammation. Moreover, inflammatory cytokine production levels and cell viability were measured in lipopolysaccharide‐responsive human embryonic kidney 293 cells (293/hTLR4A‐MD2‐CD14 cells) after fatostatin administration. In K/BxN serum‐induced arthritis mice, fatostatin treatment significantly reduced the arthritis scores and hyperplasia. *In vitro* analysis revealed that fatostatin significantly inhibited the secretion of inflammatory cytokines from cells activated with lipopolysaccharide, without affecting cell viability. This is the first study to demonstrate that fatostatin is an anti‐inflammatory agent that modulates the processing of lipid transcription factors without affecting cell viability. Accordingly, the study reveals the potential of anti‐inflammatory therapeutics that link lipid regulation and inflammation.

## Graphical abstract

Fatostatin is a small molecule that inhibits the function of sterol regulatory element‐binding protein 1. In the present study, we found that fatostatin acts as an anti‐inflammatory agent without affecting cell viability. In K/BxN serum‐induced arthritis mice, fatostatin treatment significantly reduced arthritis scores and hyperplasia. *In vitro* analysis revealed that fatostatin significantly inhibits the secretion of inflammatory cytokines from cells activated with lipopolysaccharide, without affecting cell viability.

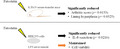

**Abbreviations**
CFSE: carboxyfluorescein succinimidyl ester
ELISA: enzyme‐linked immunosorbent assay
H&E: hematoxylin and eosin
IL: interleukin
LPS: lipopolysaccharide
LXR: liver X receptor
qRT‐PCR: quantitative real‐time PCR
S1P: site‐1 protease
S2P: site‐2 protease
SCAP: sterol regulatory element‐binding protein cleavage‐activating protein
SREBP: sterol regulatory element binding protein

The transcription factors sterol regulatory element‐binding proteins (SREBPs) and liver X receptors (LXRs) regulate fatty acid homeostasis and lipid synthesis [1]. Importantly, these transcription factors can also have antagonistic effects on immune responses [2]. The SREBP family of transcription factors is encoded by two distinct genes, *SREBF1* and *SREBF2* [3]. There are two isoforms of SREBP1, namely, SREBP‐1a and SREBP‐1c, which result from different transcription start points in *SREBF1* [4]. Regarding the regulation of lipid metabolism, SREBP2 increases cellular cholesterol synthesis [5], whereas activation of LXRs increases cholesterol efflux from macrophages [6], and overexpression of SREBP‐1a causes fatty liver *in vivo* [4]. Concerning their effects on immune responses, LXRs inhibit inflammation by reducing the activation of pro‐inflammatory transcription factors in macrophages [1], whereas SREBP1 promotes inflammation by increasing the transcription of interleukin‐1 beta (IL‐1β) [7]. However, during the resolution phase of inflammation, nuclear factor‐kappa B activation leads to a decrease in LXR expression and, instead, SREBP1 suppresses the production of pro‐inflammatory cytokines [8]. Additionally, SREBP1 upregulates the expression of omega‐3 fatty acids and 9Z palmitoleic acid regulators, which bind G‐protein coupled receptors and mediate anti‐inflammatory effects on macrophages [1, 9].

The SREBP precursor in the endoplasmic reticulum is escorted by sterol regulatory element‐binding protein cleavage‐activating protein (SCAP) to the Golgi apparatus. At its membrane binding site on the Golgi apparatus, SREBP is modified by Site‐1 protease (S1P) and Site‐2 protease (S2P), and the N‐terminus of the protein migrates to the nucleus to function as a transcription factor for the sterol regulatory element (Fig. 1) [10]. The small‐molecule fatostatin competes with SREBP for SCAP, and thus interferes with the translocation of the SREBP precursor from the endoplasmic reticulum to the Golgi apparatus, thereby preventing the maturation and function of SREBP [10, 11]. Remarkably, compared to sterols, fatostatin has a different binding site and a distinct effect on the SCAP function [11]. In addition, fatostatin inhibits cell proliferation in a lipid‐ and SCAP‐independent manner [12]. However, the ability of fatostatin to regulate inflammation is not well understood.

**Fig. 1:**
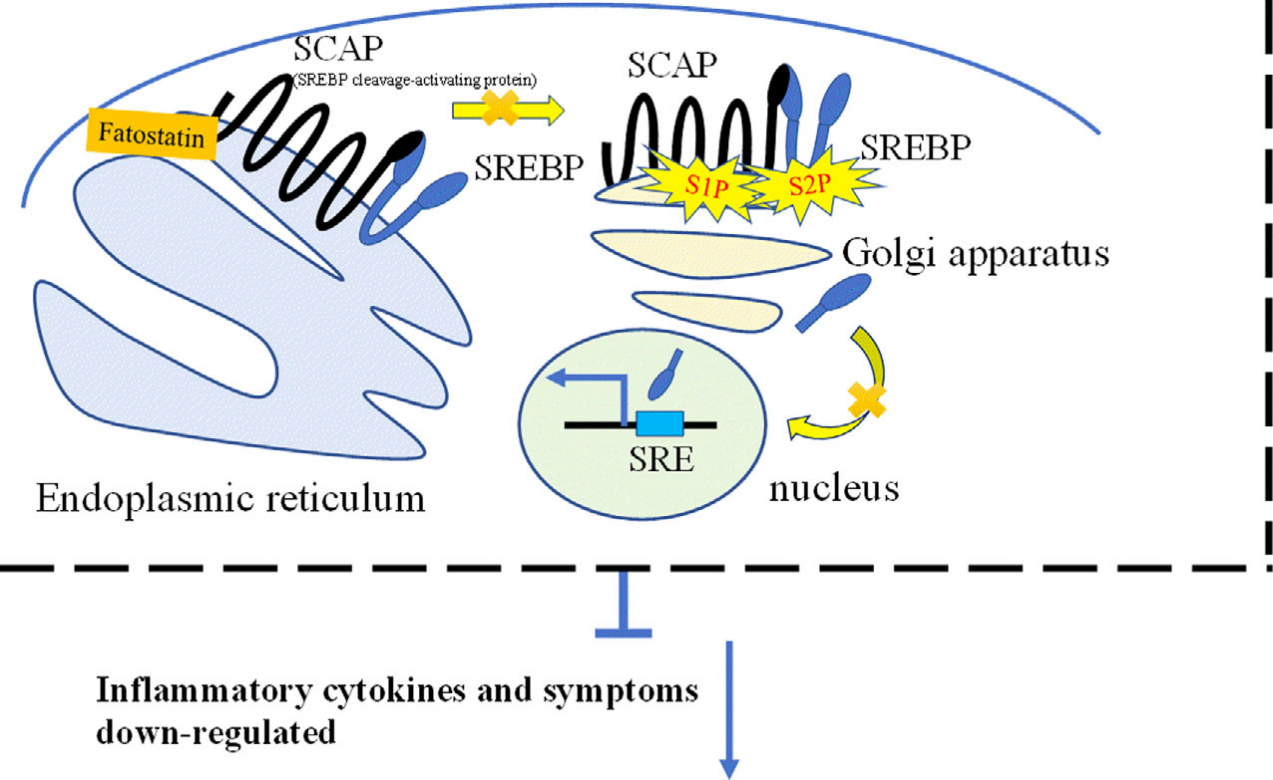
Molecular mechanism for fatostatin with respect to inhibiting production of the matured form of SREBP. Fatostatin targets SCAP and binds to a site that is distinct from the sterol‐binding domain. The SREBP precursor in the endoplasmic reticulum is translocated by SCAP to the Golgi apparatus. At its membrane‐binding site on the Golgi apparatus, SREBP is cleaved by S1P and S2P, and the N‐terminus of the protein migrates to the nucleus to function as a transcription factor that binds to the sterol regulatory element).

In the present study, we aimed to ascertain the role of fatostatin in regulating inflammation. The effect of fatostatin on inflammation was studied in a K/BxN serum‐induced arthritis mouse model. The K/BxN serum‐induced arthritis model can be triggered only with injection of serum from arthritic transgenic K/BxN mice [13, 14, 15]. Because no immunostimulatory application is needed [15], inflammation occurs more naturally. The expression levels of *Lxrb*, *Srebp1a* and *Srebp1c* of K/BxN serum‐induced arthritis mice were quantified in the presence or absence of fatostatin treatment. The scores for hematoxylin and eosin (H&E)‐stained sections of the joints from each group were compared. To determine the effect of fatostatin on cell proliferation or survival rate, we also performed *in vitro* experiments using 293/hTLR4A‐MD2‐CD14 cells, comprising HEK293 cells modified to elicit an inflammatory response to lipopolysaccharide (LPS).

## Materials and methods

### *In* *vivo* experiments for SREBP1 inhibition

#### K/BxN serum‐induced arthritis mouse model and fatostatin treatment

Six‐week‐old BALB/c male mice (*n* = 20) were used in the present study. A 14:10 h light/dark photocycle was used, and the supplementation of food and water was conducted in accordance with the instructions of Institute of Laboratory Animals, Graduate School of Medicine, Kyoto University. Food was replenished and the cage was cleaned once a week, and the water supply was provided via a nozzle in each cage set into a shelf. With a floor area of 451 cm^2^, each cage contained five mice, in accordance with the instructions of National Research Council [16]. All mice were intraperitoneally injected with 30 µL of K/BxN mouse serum, as described previously [13, 14]. The injection day was designated as day 0. Mice were then divided into two groups: control and fatostatin‐treated, and each group involved 10 mice. Mice from the control group were administered 80 µL of DMSO (#043‐07216; FUJIFILM Wako Pure Chemicals Corp, Osaka, Japan), whereas those from the fatostatin group were injected with 0.6 mg of fatostatin (#13562; Cayman, Ann Arbour, MI, USA) in 80 µL of DMSO. Both groups were administered their respective injections for 3 days. To reduce suffering, intraperitoneal injections were performed as infrequently as possible.

#### Sample collection and arthritis score evaluation

From days 0 to 10, mice were weighed, their footpad and hock dimensions were measured, and arthritis scores were defined as 0 = no inflammation, 1 = minimal inflammation, 2 = mild inflammation, 3 = moderate inflammation and 4 = severe inflammation using the method defined by previous studies [17, 18]. On day 12, mice were killed by cervical dislocation, and splenic F4/80 positive cells, blood plasma and the distal limbs were collected from each mouse.

Spleens of mice were homogenized using a 70‐µm cell strainer and 2‐mL syringe plungers, and thereafter centrifuged at 620 ***g*** for 3 min. The cell pellet was washed twice with Hanks' balanced salt solution (#14170112; Thermo Fisher, Waltham, MA, USA). To obtain F4/80 positive cells, the pellets were dissolved in a solution of 0.5 m EDTA in phosphate‐buffered saline containing 0.1% BSA and centrifuged at 300 ***g*** for 10 min before being subjected to magnetic activated cell sorting, with anti‐F4/80 MicroBeads UltraPure (#130‐110‐443; Miltenyi Biotec, Bergisch Gladbach, Germany). Distal limbs of the mice were detached using animal surgery scissors for phenotypic analysis via H&E staining of sections. Blood was collected by cardiac blood sampling and then centrifuged at 1200 ***g*** for 30 min at 4 °C to isolate the plasma. Triglyceride and cholesterol concentrations in the blood plasma of mice were measured by SRL, Inc. (Tokyo, Japan).

### *In* *vitro* experiments for fatostatin administration

The 293/null cells (#293‐null; InvivoGen, San Diego, CA, USA), HEK293 cells and 293/hTLR4A‐MD2‐CD14 cells (#293‐htrl4md2cd14; InvivoGen), comprising HEK293 cells modified to respond to LPS, were cultured in growth medium composed of Dulbecco's modified Eagle’s medium/nutrient mixture F‐12 (#21331‐046; Gibco, Waltham, MA, USA) containing 10% (v/v) fetal bovine serum, 50 U·mL^−1^ penicillin, 50 µg·mL^−1^ streptomycin (penicillin/streptomycin 100×, #15070; Gibco), 15 mm Hepes (#15630; Gibco) and 2 mm l‐glutamine (Glutamax 100×, #35050; Gibco) and then incubated at 37 °C in 5% CO_2_ for 4 days. To maintain the plasmid coding for hTLR4a and MD2‐CD14, the growth medium for 293/hTLR4A‐MD2‐CD14 cells was supplemented with 10 µg·mL^−1^ blasticidin and 50 µg·mL^−1^ hygromycin B gold [19].

### Experimental methods

#### Quantitative real‐time polymerase chain reaction

RNA was isolated using Isogen (#315‐02504; Nippon Gene, Tokyo, Japan) and the RNeasy Mini kit (#74104; Qiagen, Hilden, Germany). After DNase treatment (#M6101; Promega, Madison, WI, USA), the complementary DNA was prepared using an iScript cDNA Synthesis Kit (#1708890; Bio‐Rad, Hercules, CA, USA), in accordance with the manufacturer’s instructions. A quantitative real‐time PCR (qRT‐PCR) was performed using TB Green (Takara, Kusatsu, Japan) in a 7500 Real‐Time PCR System (Applied Biosystems, Waltham, MA, USA). Conditions for qRT‐PCR were: one cycle for the initial denaturation stage at 95 °C for 30 s, 70 cycles for the PCR stage with denaturation at 95 °C for 10 s and annealing at 60 °C for 34 s, and the setting for the melt curve stage was in accordance with the instructions of the Takara TB Green for 7500 Real‐Time PCR System. qRT‐PCR primers for mouse samples are listed in Table 1 [20, 21, 22].

**Table 1:** Primers sequences for running quantitative real‐time polymerase chain‐reaction.

Gene	Forward Sequences (5′‐ to 3′)	Reverse sequence (5′‐ to 3′)	Reference
Mouse			
*Rpl13a*	GCTCTCAAGGTTGTTCGGCTGA	AGATCTGCTTCTTCTTCCGATA	[20]
*Srebp1a*	TAGTCCGAAGCCGGGTGGGCGCCGGCGCCAT	GATGTCGTTCAAAACCGCTGTGTGTCCAGTTC	[21]
*Srebp1c*	ATCGGCGCGGAAGCTGTCGGGGTAGCGTC	ACTGTCTTGGTTGTTGATGAGCTGGAGCAT	[21]
*Nr1h2* (*Lxrb*)	GCCTGGGAATGGTTCTCCTC	AGATGACCACGATGTAGGCAG	[22]

### Western blotting

293/hTLR4A‐MD2‐CD14 cells were resuspended at 10^6^·mL^−1^ in growth medium and cultured with 0, 10 or 20 µm fatostatin in 0.5% DMSO for 44 h before collecting cell lysate with radioimmunoprecipitation assay buffer (#08714‐04; Nacalai Tesque, Kyoto, Japan). The protein concentration was measured using a BCA protein assay kit (#23227; Thermo Scientific) at 562 nm absorbance in conjunction with a NanoDrop 1000 Spectrophotometer (Thermo Scientific). To measure the SREBP1 levels in the cytoplasm, 12 μg of protein was applied for each sample and 5 μg of protein was applied for each sample to measure the levels of beta‐actin in the cytoplasm. The gel was run for 45 min at 220 V. The membrane was washed six times with 1 × Tris‐buffered saline with 0.1% Tween^®^ 20 detergent (0.05% Tween 20 in 1 × Tris‐buffered saline) and incubated with Bullet Blocking One for western blotting (#13779‐14; Nacalai Tesque) at 23 °C–25 °C for 5 min. After washing the membrane six times, it was incubated with primary antibodies (SREBP1: clone, 2A4, #MA5‐16124; Thermo Fisher; beta‐actin: clone, 8H10D10, #3700; Cell Signaling Technology, Danvers, MA, USA), which were diluted 500 times and 15 000 times in Blocking One solution (#03953‐95; Nacalai Tesque) at 23–25 °C for 2–2.5 h with rotation. After six washes, the membrane was incubated with a conjugated secondary antibody diluted 3000 times and 10 000 times in Blocking One solution (#03953‐95; Nacalai Tesque) at 23–25 °C for 40 min. After six washes, the membrane was developed using SuperSignal (#34577; Thermo Scientific) for 5 min and exposed to ultraviolet light for 180 s using a ChemiDoc MP system (Bio‐Rad). Images were analyzed using image lab, version 4.0 software (Bio‐Rad) and imagej (NIH, Bethesda, MD, USA).

### Cell proliferation assay

For the proliferation assay with 1.5 µm carboxyfluorescein succinimidyl ester (CFSE) (#150347‐59‐4; Dojindo, Rockville, MD, USA), 293/null cells were resuspended in growth medium at a density of 10^6^·mL^−1^ and cultured in six‐well plates with 0, 10 or 20 µm fatostatin. CFSE‐treated cells that were cultivated in the absence of DMSO or fatostatin were used as negative controls. Cells were labeled with CFSE in accordance with the manufacturer’s instructions (#13‐0850‐U50; TONBO Biosciences, San Diego, CA, USA). The 293/null cells were incubated at 37 °C in 5% CO_2_ and harvested 44 h after fatostatin treatment. Cells were washed once with 1 × phosphate‐buffered saline and 1 × binding buffer (#00‐0055; Invitrogen, Waltham, MA, USA) each, at 620 ***g*** for 3 min. The pellet was resuspended in 200 µL of 1 × binding buffer, and incubated on ice for 15 min after the addition of 5 µL of 7‐AAD Viability Staining Solution (#559925; BD, Franklin Lakes, NJ, USA). Cell divisions were measured using a LSRFortessa cytometer (BD) and data were analyzed using flowjo, version 10.4 (BD).

### Enzyme‐linked immunosorbent assay (ELISA)

Before being incubated with 0, 10 or 20 µm fatostatin for 17 h, the 293/hTLR4A‐MD2‐CD14 cells were resuspended in growth medium at a concentration of 8 × 10^5^·mL^−1^ and cultured for 48 h in 48‐well plates. Cells were then treated with the TLR4 agonist LPS (# L4524‐5MG; Sigma, St Louis, MO, USA) for 24 h. Cells cultivated in the absence of DMSO or fatostatin were used as negative controls. Supernatants of 293/hTLR4A‐MD2‐CD14 cells were used for an ELISA (#431501; BioLegend, San Diego, CA, USA) for IL‐8 in accordance with the manufacturer’s instructions. Absorbance was measured at 450 nm using the iMark microplate reader (Bio‐Rad) and data were analyzed using Microplate manager 6 software (Bio‐Rad). Cells were stained with trypan blue and counted with a Countess‐Automated Cell Counter (Invitrogen) to evaluate the total, live and dead numbers. Survival rates were calculated as the percentage of cells alive after fatostatin treatment.

### Statistical analysis

*P* < 0.05 was considered statistically significant. Data for animal weight, footpad and ankle thickness, as well as for arthritis scores, are presented as the mean ± SD. Statistical analysis for the H&E‐stained sections was performed using Mann–Whitney *U*‐tests and Fisher’s exact tests. The 2^−ΔΔCT^ method was used to compare the qRT‐PCR data. The Mann–Whitney *U*‐test was used to analyze the statistical differences in the *in vitro* study. Error bars are defined as the mean ± SD. Statistical analyses were performed using jmp pro, version 15.2 (SAS Software, Cary, North Carolina, USA) and origin, version 9.0 (Originlab, Northampton, MA, USA).

### Ethical declaration

The study was approved by the Animal Research Committee of the Graduate School of Medicine, Kyoto University, Kyoto, Japan (MedKyo 20115). All experimental procedures were performed in accordance with the ethical guidelines of the Kyoto University. The study was performed following COPE guidelines and was in compliance with ARRIVE guidelines 2.0 (https://arriveguidelines.org/arrive‐guidelines).

## Results

### Fatostatin has beneficial effects on mice with K/BxN serum‐induced arthritis

**Fig. 2:**
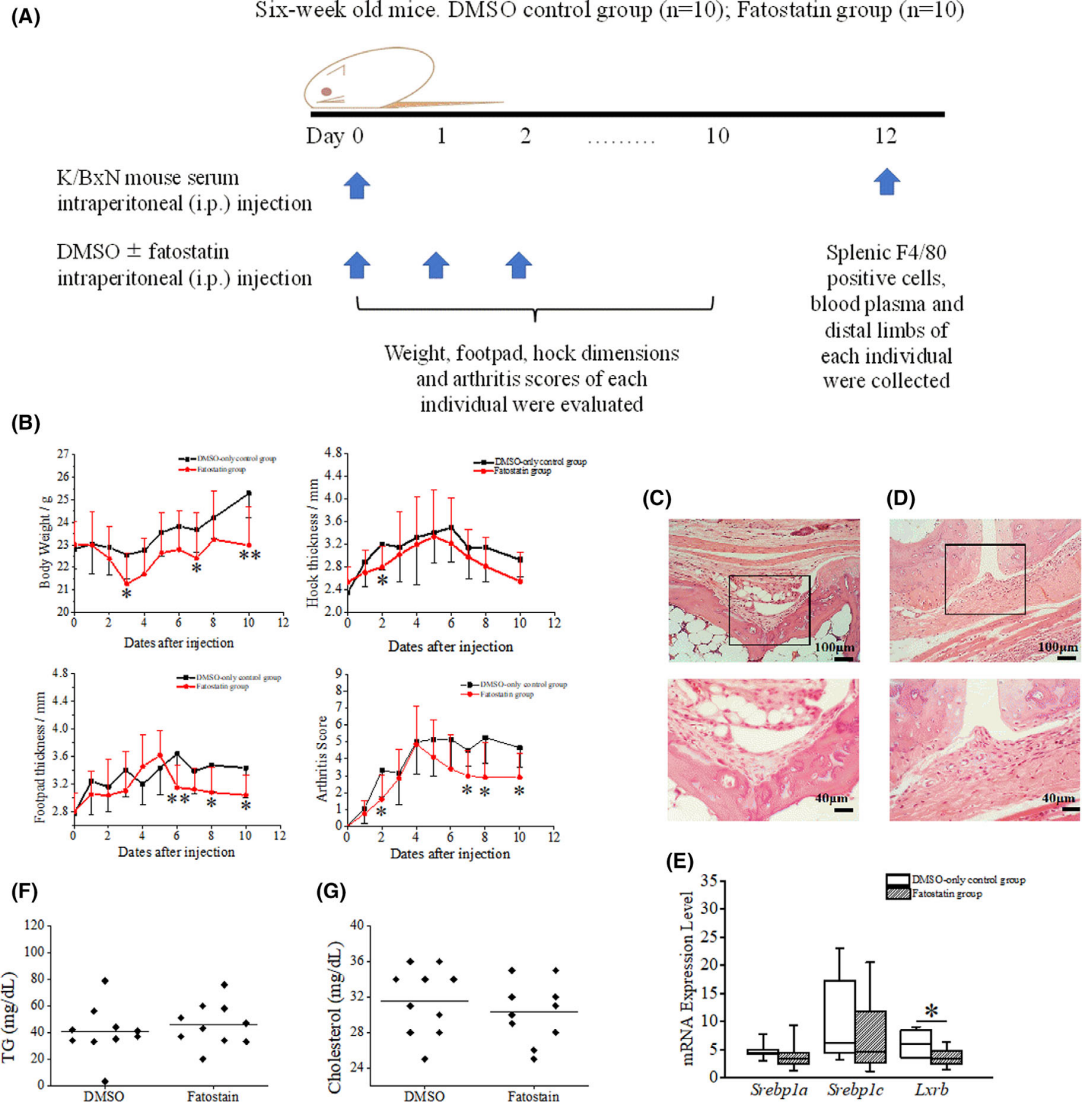
Fatostatin treatment has beneficial effects on mice with K/BxN serum‐induced arthritis. (A) Schematic for fatostatin administration and sample collection in KxB/N serum‐induced mice. Six‐week‐old BALB/c male mice were intraperitoneally injected with 30 µL of K/BxN mouse serum on day 0. From day 0 onwards, the control group was administered 80 µL of DMSO and the fatostatin group (*n* = 10 per group) was injected with a solution of 0.6 mg fatostatin in 80 µL of DMSO, for 3 days every day. From days 0 to 10, the body weight, dimensions of footpads and hocks, and arthritis scores of each mouse were evaluated. On day 12, splenic F4/80 positive cells, blood plasma and distal limbs were collected from each mouse. (B) Changes in body mass, footpad thickness, hock thickness and arthritis scores after fatostatin or DMSO injections (mean ± SD, *n* = 10 per group). *p* = 0.0355, 0.0232 and 0.0029 for the body weights on days 3, 7 and 10, respectively; *p* = 0.0303 for the hock thickness on day 2; *p* = 0.0014, 0.0449 and 0.0174 for the footpad thickness on days 6, 8 and 10, respectively; *p* = 0.0234, 0.0101, 0.0081 and 0.0153 for the arthritis score between fatostatin‐treated and untreated groups on days 2, 7, 8 and 10, respectively. Arthritis scores were assigned for each paw: 0 = no inflammation, 1 = minimal inflammation, 2 = mild inflammation, 3 = moderate inflammation and 4 = severe inflammation. Error bars indicate the mean ± SD. *P < 0.05 and **P < 0.01 as determined by Mann–Whitney *U*‐tests. (C, D) Representative H&E stained distal paw sections obtained on day 12 after the first 3 days of DMSO (C) or fatostatin (D) intraperitoneal injection. Upper and lower images were from the same samples, and the outlined part has been enlarged 2.5 times in the lower images. Arrows indicate lymphocytes. (E) mRNA expression levels of *Srebp1a*, *Srebp1c* and *Lxrb* in DMSO (*n* = 10) and fatostatin (*n* = 10)‐treated mice. The *y*‐axis indicates the relative expression levels, with *Rpl13a* as the housekeeping gene. *P < 0.05 as determined by Mann–Whitney *U*‐tests. (F, G) Comparison of blood plasma triglyceride and cholesterol levels between the DMSO control and the fatostatin group.

Figure 2A shows a schematic diagram for fatostatin treatment and sample collection from K/BxN mice. Briefly, K/BxN serum and fatostatin were both administered via intraperitoneal injections. From days 0 to 10, body weight, footpad and hock dimensions, and arthritis scores of each mouse were evaluated. On day 12, splenic F4/80 positive cells, blood plasma and distal limbs were collected from each mouse. The arthritis scores on days 7, 8 and 10 after serum transfer (*P* = 0.0101, 0.0081 and 0.0153, respectively) (Fig. 2B) for mice from the fatostatin group were significantly lower than those of mice from the control group, which were administered only DMSO. The arthritis scores were assigned according to the scoring method defined in previous studies [17, 18]. The histology of wild‐type mice before arthritis induction can be found in a prior study [23], although representative histological analysis with H&E‐staining also revealed severe hyperplasia in the DMSO‐treated group (Fig. 2C) compared to the fatostatin‐treated group (Fig. 2D). Although lymphocyte infiltration did not demonstrate any significance between the two groups (Table 2), different infiltration patterns can be seen in Fig. 2C,D, where examples of lymphocyte models were provided in the previous literature [24, 25]. Notably, all mice in the control group developed hyperplasia, whereas four mice from the fatostatin group did not (*P* = 0.0325, Fisher’s exact test) (Table 2).

**Table 2:** Mouse hind paw arthritis scores, determined using H&E‐stained sections. ‘N’ refers to DMSO‐treated mice and ‘F’ refers to fatostatin‐treated mice. The scores were defined as: 0 = no inflammation, 1 = minimal inflammation, 2 = mild inflammation, 3 = moderate inflammation and 4 = severe inflammation. ‘1’ and ‘0’ indicate whether hyperplasia, cell infiltration into the bone marrow and vessels in stroma were observed or not in each H&E‐stained section. *P* values for lymphocyte infiltration and fibrosis score were determined by Mann–Whitney *U*‐tests. The *P* value for hyperplasia, cell infiltration into the bone marrow and vessels in stroma were determined by Fisher’s exact test. Bold indicates statistical significance (*P* < 0.05).

	Lymphocyte infiltration	Fibrosis score	Lining hyperplasia	Bone marrow infiltration	Vessels in stroma
N1	2	2	1	0	1
N2	1.5	1.5	1	1	1
N3	1	2	1	0	1
N4	0.5	0	1	0	1
N5	1	0	1	0	1
N6	1.5	1	1	1	1
N7	2.5	1	1	1	1
N8	4	0.5	1	0	1
N9	3	3	1	0	1
N10	2	0.5	1	0	1
Mean	1.9	1.15	1	0.3	1
SD	0.9950	0.9233	0	0.4583	0
F1	0.5	0.5	1	0	1
F2	0	0.5	0	0	1
F3	2	1.5	1	0	1
F4	0	0.5	0	0	1
F5	2	0.5	0	0	1
F6	2	0	0	0	1
F7	3	0	1	0	1
F8	Not measured				
F9	3	1	1	1	1
F10	3	0	1	0	1
Mean	1.7222	0.5000	0.5556	0.1111	1
SD	1.1811	0.4714	0.4970	0.3142	0
*P* value (between N and F)	0.9669	0.1309	**0.0325**	0.5820	1

To determine whether fatostatin affected the expression of key transcription factors related to lipid regulation, their expression in splenic F4/80 positive cells was monitored via qRT‐PCR analysis. Moreover, *Lxrb* (*Nr1h2*) expression was significantly downregulated in the fatostatin group (*P* = 0.0288) (Fig. 2E) and *Srebp1a* and *Srebp1c* were expressed at lower levels in the fatostatin‐treated group, although a significant difference was not detected among them (*P* = 0.2177 and 0.3527, respectively). However, fatostatin did not affect plasma triglyceride or cholesterol concentrations (Fig. 2F,G).

### Fatostatin reduces cell proliferation in a dose‐dependent manner

**Fig. 3:**
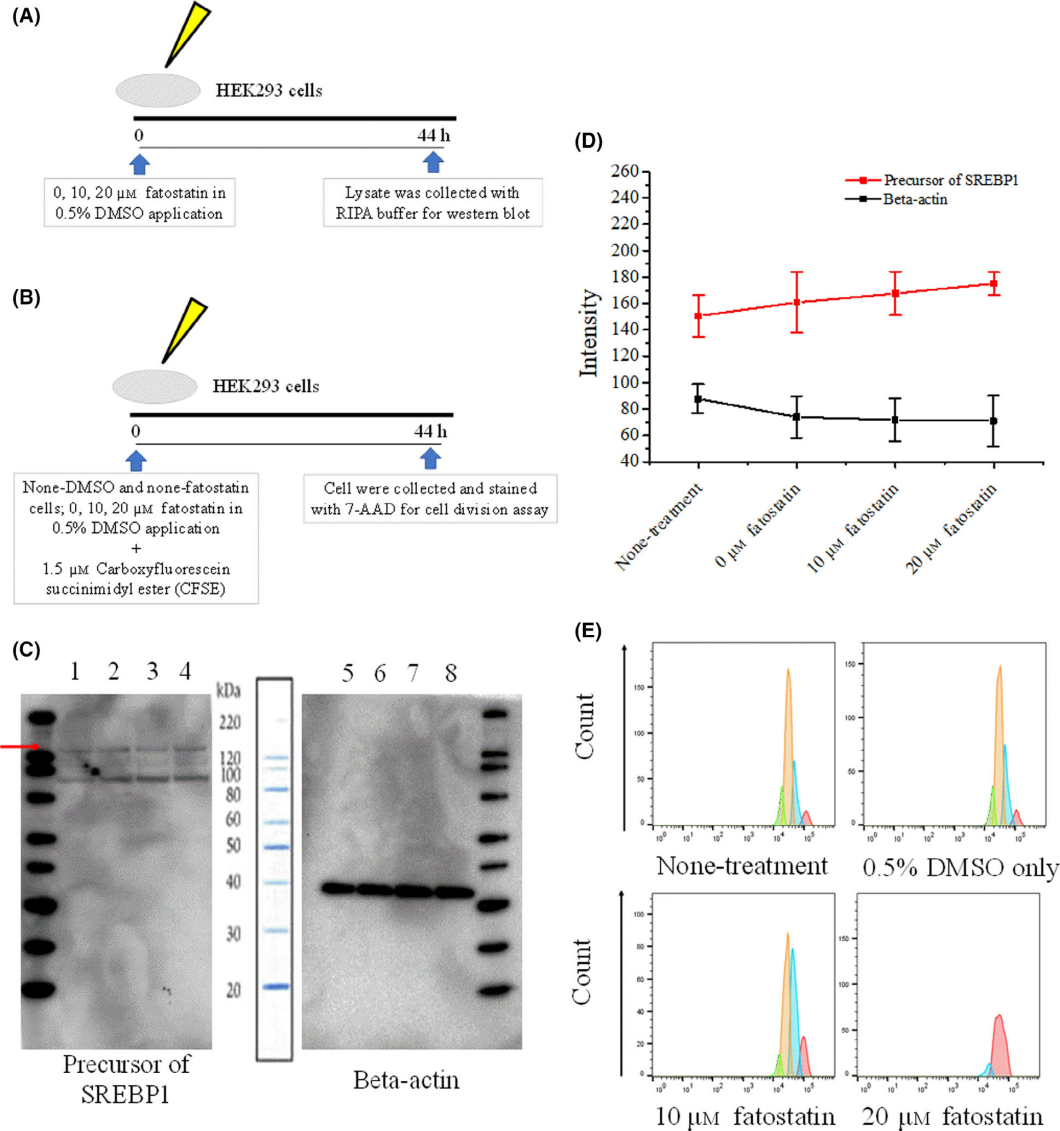
Fatostatin reduces cell proliferation in a dose‐dependent manner. (A, B) Schematic for western blotting and CFSE cell division assays for fatostatin treatment in HEK293 cells. Cell lysates or cells were collected 44 h after fatostatin treatment. (C) Representative western blot of precursor SREBP1 (125 kDa) measured in HEK293 cells treated by fatostatin. Lines 1, 2, 3 and 4 indicate SREBP1 precursor protein levels in the cytoplasm of HEK293 cells, and the cells were cultred without DMSO (Line 1) and culterd with 0 (Line 2), 10 μm (Line 3) and 20 μm (Line 4) fatostatin (in 0.5% DMSO) for 44 h, respectively. The red arrow indicates the line of the bands assessed for SREBP expression and activity. Lines 5, 6, 7 and 8 indicate beta‐actin protein levels in the cytoplasm of HEK293 cells, and the cells were cultred without DMSO (Line 1) and culterd with 0 (Line 2), 10 μm (Line 3) and 20 μm (Line 4) fatostatin (in 0.5% DMSO) for 44 h, respectively. (D) Quantification of western blot intensity results. Data are the mean ± SD of four individual measurements. The intensity is defined by imagej. (E) Cell division assay for HEK293 fatostatin‐treated cells. Red, blue, yellow and green histograms represent divisions 0, 1, 2 and 3, respectively.

Because fatostatin‐treated mice showed lower levels of hyperplasia compared to the DMSO‐treated mice, we next determined whether fatostatin treatment could affect cell division. Using LPS‐responsive HEK293 cells, we investigated whether fatostatin can improve inflammation in cells expressing SREBPs in addition to monocytes and macrophages. To confirm whether fatostatin inhibits SREBP maturation, aliquots of cell lysates from HEK293 cells, which were cultured with 0, 10 and 20 μm fatostatin (in 0.5% DMSO) for 44 h, were used for western blot analysis (Fig. 3A). The other cells cultured under the same conditions as for the western blot were treated with CFSE for the cell proliferation assay (Fig. 3B). The SREBP1 antibody used in the western blot targets both the mature form (60–70 kDa) and the precursor form (125 kDa) of SREBP1 and we measured and quantified the bands at 125 kDa in four individual experiments. As shown in Fig. 3C,D, an increase in the concentration of fatostatin was associated with an increase in the intensity of the bands for the SREBP precursor, indicating that fatostatin reduced the maturation of SREBP. This was also confirmed by another study in which fatostatin was shown to block lipid synthesis by inhibiting the activation of SREBP [11]. To determine the effect of fatostatin on cell proliferation, CFSE analysis of HEK293 cells treated with different concentrations of fatostatin was performed (Fig. 3E). Red, blue, yellow and green histograms represent 0, 1, 2 and 3 divisions, respectively. Evidently, fatostatin treatment reduced cell proliferation in a dose‐dependent manner.

### Fatostatin treatment suppresses inflammation without affecting cell viability

**Fig. 4:**
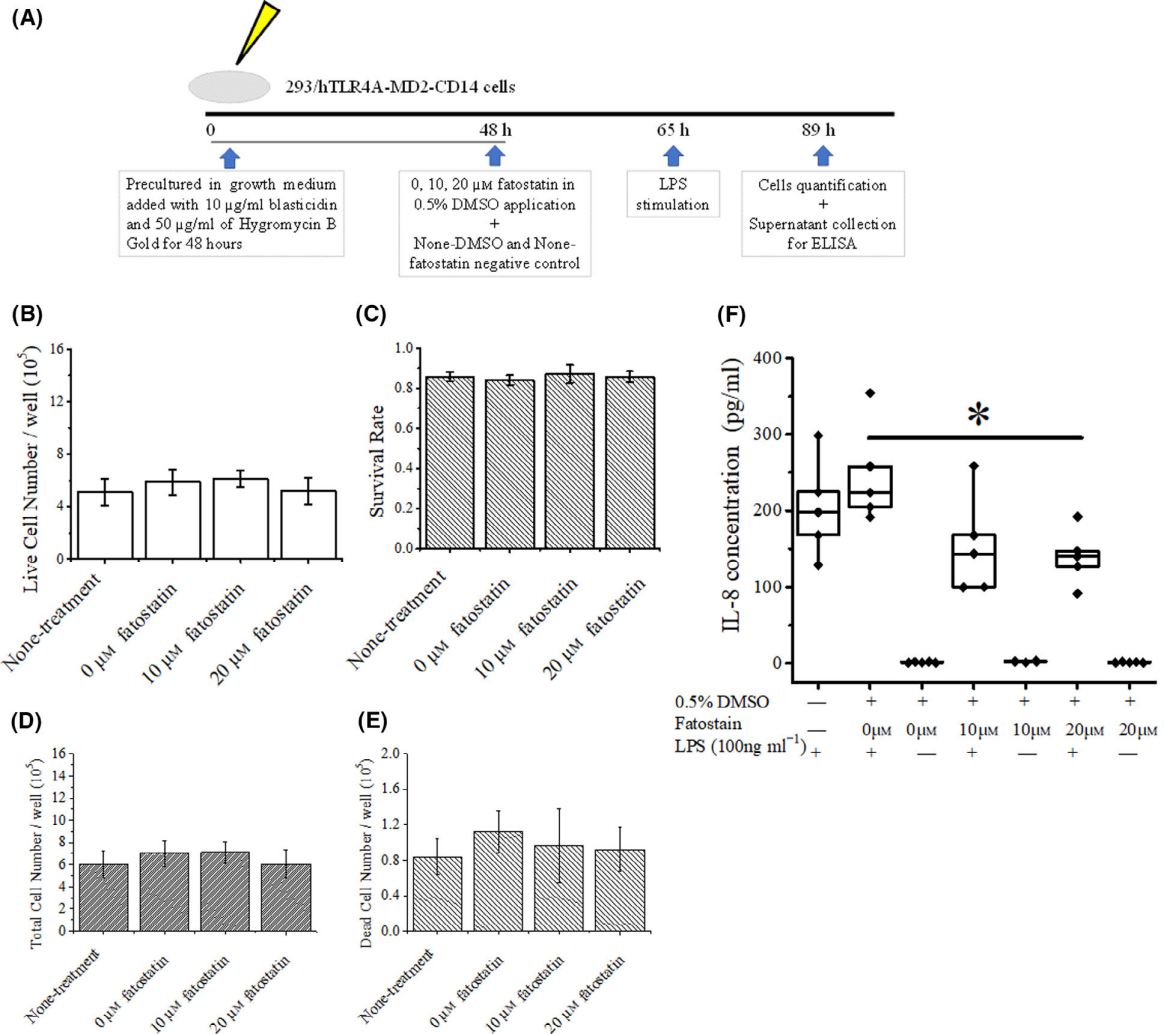
Treatment with fatostatin suppresses inflammation without affecting cell survival rate. (A) Schematic for the ELISA of fatostatin‐treated 293/hTLR4A‐MD2‐CD14 cells. Growth medium was composed of Dulbecco's modified Eagle’s medium/nutrient mixture F‐12 supplemented with 10% (v/v) fetal bovine serum, 50 U·mL^−1^ penicillin, 50 µg·mL^−1^ streptomycin, 15 mm Hepes and 2 mm l‐glutamine. Plasmids coding for hTLR4a and MD2‐CD14 were maintained by supplementing the growth medium with 10 µg·mL^−1^ blasticidin and 50 µg·mL^−1^ hygromycin B gold. (B, C) The numbers of living cells and survival rates of the cells from each of the fatostatin‐treated group. (D, E) The numbers of total cells and dead cells from each of the fatostatin‐treated group. (F) Concentration of IL‐8 in each fatostatin‐treated group with or without LPS. *P* = 0.0216 between the DMSO‐only and 20 μm fatostatin‐treated groups. Cells were pre‐treated with different concentrations of fatostatin for 17 h and then stimulated with or without LPS for 24 h. Error bars indicate the mean ± SD. *P < 0.05 was determined by Mann–Whitney *U*‐tests.

Next, we investigated the mechanism by which fatostatin improves inflammation in arthritis via 293/hTLR4A‐MD2‐CD14 cell culture experiments. Some 17 h after the addition of fatostatin, each group of cultured cells was stimulated with LPS (time 65 h in Fig. 4A). As shown in Fig. 4B,C, after 24 h of LPS stimulation (time 89 h in Fig. 4A), no differences were observed with respect to the number of living cells or the cell survival rate in each group. The numbers of total and dead cells in each group are shown in Fig. 4D,E. Activation of cells by LPS was evaluated by IL‐8 production (Fig. 4F). The median (interquartile range) of IL‐8 concentration in the 0 μm fatostatin pretreated group was 224.23 (198.05–306.21) pg·mL^−1^, whereas it was significantly decreased to 139.85 (109.26–169.86) pg·mL^−1^ in the 20 μm fatostatin pretreated group (*P* = 0.0216). Because the production of IL‐8 was not detected in cells treated with fatostatin in the absence of LPS, it was considered that fatostatin treatment alone could not induce inflammation.

## Discussion

The present study suggests that treatment with fatostatin ameliorated inflammation in K/BxN serum‐induced arthritis mice. Our *in vitro* experiments demonstrated that fatostatin treatment significantly reduced the release of IL‐8 and contributed to the amelioration of inflammation without affecting cell viability.

The inflammatory roles of SREBP1 and SREBP2 in macrophages have been reported elsewhere [1, 26, 27] and our study has demonstrated that the severity of inflammation in joints was decreased in K/BxN mice treated with fatostatin, as shown in Fig. 2B. However, the mechanism shown in Fig. 1 is only observed in the cultured cell models and does not necessarily reflect the results of the animal model used in the present study. Although the results of Fig. 2B and Table 2 may contradict each other with respect to the significance of the improvement, Table 2 indicates a localized change compared to that of Fig. 2B and may not reflect the average outcome. In H&E staining, lymphocyte infiltration was not affected by fatostatin, probably as a result of its non‐rigorous quantitative nature, although the significantly ameliorated hyperplasia highlighted the effects of fatostatin in inflammation.

To further evaluate the role of fatostatin in inflammation, we performed *in vitro* experiments using 293/hTLR4A‐MD2‐CD14 cells, comprising HEK293 cells modified to respond to LPS. Regarding the influence of fatostatin on cell division, the results shown in Fig. 3E contradict those of Fig. 4B. However, it is plausible that, during the 48 h of pre‐cultivation for the ELISA experiments (time 0–48 h in Fig. 4A), cell proliferation was just before the plateau, such that the fatostatin‐dependent suppression of cell division may not have an effect on cell number. Thus, it can be suggested that inhibition of the mature form of SREBP with certain concentrations of fatostatin suppresses inflammation without disturbing cell proliferation. On the other hand, fatostatin significantly reduced the expression of *Lxrb* in splenic F4/80 positive cells from K/BxN mice (Fig. 2E), without affecting triglyceride and cholesterol levels in the blood plasma (Fig. 2F,G). A plausible explanation for this observation may be that the blood plasma levels of triglycerides and cholesterol were conserved as a result of homeostasis. In splenic F4/80 positive cells, the downregulation of *Lxrb* expression may be associated with that of *Srebp1c* because SREBP1C can be directly activated by LXRs [28]. However, the precise mechanism remains to be clarified.

It has been demonstrated that fatostatin can inhibit the activation of both SREBP1 and SREBP2, which belong to the SREBP family [11]. SREBP2 has been reported to cause an increase in the synthesis of cellular cholesterol [5] and was shown to bind inflammatory and interferon response target genes to promote inflammation [26]. Because our qRT‐PCR data on SREBP2 analysis in a previous study (unpublished data but contributed by Shuhe Ma) did not demonstrate any significant expression, the levels of the SREBP1 precursor were measured by western blotting (Fig. 3C,D) and we observed an increase of the SREBP1 precursor in the cytoplasm after fatostatin treatment. It should be noted that, because no induction of SREBP1 precursor was involved, the precursor levels were not significantly increased as a result of inhibition of endoplasmic reticulum‐to‐Golgi translocation or by S1P/S2P downstream proteolytic cleavage as a result of fatostatin application, which was consistent with our findings shown in Fig. 3D. We also tested *IL‐6* and *CXCL10* expression levels in splenic macrophages other than *SREBP1A*, *SREBP1C* and *LXRb* (data not shown); however, they did not show any significant differences compared to the DMSO group. This might be because fatostatin specifically functioned on cells in certain kinds of inflammatory environment *in vivo*.

The present *in vitro* study further demonstrates that fatostatin ameliorates inflammation without affecting cell viability. IL‐8 was tested because IL‐8 is a better nuclear factor‐kappa B activation indicator than IL‐6 in HEK293, and the response to TLR4 stimulation in 293/hTLR4A‐MD2‐CD14 cells can be satisfactorily evaluated by IL‐8. However, we cannot exclude the possibility that the off‐target effects of fatostatin have not eliminated in the present study; thus, experiments utilizing *Srefp1* knockout mice, as well as those evaluating the effects of fatostatin on inflammation of human arthritic tissue, should be performed in future studies.

## Conclusions

Treating K/BxN arthritis mice with fatostatin leads to an amelioration of inflammation. Furthermore, fatostatin caused a significant reduction in inflammatory cytokine production in LPS‐responsive HEK293 cells. The constant cell survival rate between the fatostatin‐treated and non‐treated groups suggests its potential role as an anti‐inflammatory agent, which links lipid regulation and inflammation.

## Conflict of interests

The authors declare that they have no conflicts of interest.

## Author contributions

SM and KM were responsible for the study design. SM and KT performed the experiments. SM wrote the first draft of the manuscript. MH, MT, KK, SA, RN, HY, KO, AM and TM supervised the draft of the manuscript. All of the authors approved the final version of the manuscript submitted for publication.

## Data Availability

The authors confirm that all of the data supporting the findings of the present study are available within the article.
